# Relationship between breast cancer risk factors and mammographic breast density in the Fernald Community Cohort

**DOI:** 10.1038/bjc.2012.1

**Published:** 2012-01-26

**Authors:** L Yaghjyan, M C Mahoney, P Succop, R Wones, J Buckholz, S M Pinney

**Affiliations:** 1 Department of Environmental Health, University of Cincinnati College of Medicine, PO Box 670056, Cincinnati, OH 45267, USA; 2 Department of Radiology, University of Cincinnati College of Medicine, 234 Goodman Avenue, Cincinnati, OH 45267, USA; 3 Department of Internal Medicine, University of Cincinnati College of Medicine, 231 Albert B. Sabin Way, Room 6065, PO Box 670557, Cincinnati, OH 45267, USA

**Keywords:** breast density, breast cancer risk factors, risk modification

## Abstract

**Background:**

We investigated associations of known breast cancer risk factors with breast density, a well-established and very strong predictor of breast cancer risk.

**Methods:**

This nested case–control study included breast cancer-free women, 265 with high and 860 with low breast density. Women were required to be 40–80 years old and should have a body mass index (BMI) <35 at the time of the index mammogram. Information on covariates was obtained from annual questionnaires.

**Results:**

In the overall analysis, breast density was inversely associated with BMI at mammogram (*P* for trend<0.001), and parity (*P* for trend=0.02) and positively associated with alcohol consumption (ever *vs* never: odds ratio 2.0, 95% confidence interval 1.4–2.8). Alcohol consumption was positively associated with density, and the association was stronger in women with a family history of breast cancer (*P*<0.001) and in women with hormone replacement therapy (HRT) history (*P*<0.001). Parity was inversely associated with density in all subsets, except premenopausal women and women without a family history. The association of parity with density was stronger in women with HRT history (*P*<0.001).

**Conclusion:**

The associations of alcohol and parity with breast density appear to be in reverse direction, but stronger in women with a family history of breast cancer and women who ever used HRT.

Mammographic breast density is a strong predictor of breast cancer with odds ratios (ORs) varying from 1.8 to 6.0 (Henderson and Feigelson, 2000; Ursin *et al*, 2003; Boyd *et al*, 2006; Martin *et al*, 2006; Tamimi *et al*, 2007; Vachon *et al*, 2007; Ginsburg *et al*, 2008; Ghosh and Vachon 2010; Kerlikowske *et al*, 2010). Mammographic breast density is a reflection of the amount of fat, connective, and epithelial tissue in the breast (Warren, 2004). Light (non-radiolucent or dense) areas on the mammogram represent the fibrous and glandular tissues in the breast, whereas the dark (radiolucent or less dense) areas are primarily fat. The degree of density is a consequence of the hormonal environment and underlying genetics regulating the epithelial proliferation (Warren, 2004).

Several studies have evaluated predictors of breast density in women who have not been diagnosed with breast cancer. Inverse associations with breast density were reported for older age, parity, postmenopausal status, and high body mass index (BMI) (El-Bastawissi *et al*, 2000; Lam *et al*, 2000; Vachon *et al*, 2000; Colacurci *et al*, 2001; Erel *et al*, 2001; Vachon *et al*, 2002; Gapstur *et al*, 2003; Warwick *et al*, 2003; Conner *et al*, 2004; Vachon *et al*, 2005; Maskarinec *et al*, 2006; Modugno *et al*, 2006; Titus-Ernstoff *et al*, 2006; Ginsburg *et al*, 2008; Johansson *et al*, 2008; Kelemen *et al*, 2008). A positive association between age at menarche and density was reported to be stronger in premenopausal women (El-Bastawissi *et al*, 2000; Vachon *et al*, 2000); in some studies (Vachon *et al*, 2000) these associations were attenuated in multivariate analyses. Hormone replacement therapy (HRT), older age at first child's birth, nulliparity, a family history of breast cancer, and alcohol consumption have been reported to be associated with increased breast density (Gram *et al*, 1995; El-Bastawissi *et al*, 2000; Vachon *et al*, 2000, 2002, 2005; Colacurci *et al*, 2001; Erel *et al*, 2001; Rutter *et al*, 2001; Gapstur *et al*, 2003; Ziv *et al*, 2003; Conner *et al*, 2004; Harvey *et al*, 2005; Boyd *et al*, 2006; Maskarinec *et al*, 2006; Titus-Ernstoff *et al*, 2006). Inconsistent relations with breast density have been reported for smoking, circulating hormone levels (blood estrogen, estrone levels, total estradiol levels, sex hormone-binding globulin), race/ethnicity, and a few polymorphisms in the estrogen metabolism pathway genes (Boyd *et al*, 2002; Haiman *et al*, 2002; Gapstur *et al*, 2003; Warwick *et al*, 2003; Conner *et al*, 2004; Aiello *et al*, 2005; Noh *et al*, 2006; Warren *et al*, 2006; Bremnes *et al*, 2007; Maskarinec *et al*, 2007; Tamimi *et al*, 2007; Verheus *et al*, 2007). Whether the associations of breast cancer risk factors with breast density are modified by other characteristics is poorly understood. Only a few studies have examined associations of breast cancer risk factors with breast density in different subsets of women and reported on interactions between a few breast cancer risk factors (Boyd *et al*, 2002; Gapstur *et al*, 2003; El-Bastawissi *et al*, 2005; Aiello *et al*, 2006). To further investigate predictors of high breast density, we evaluated a range of breast cancer risk factors and assessed whether these associations differ in the subsets of women defined on the basis of menopausal status, HRT history, or a family history of breast cancer. We report for the first time statistically significant interactions of parity and alcohol use with HRT, as well as interaction of alcohol use with a family history of breast cancer. Finally, in contrast to all previous studies, we defined the breast density phenotype based on the life-long density history for each woman rather than a single mammogram.

## Materials and methods

### Study population

This study was conducted using mammograms and the data available from an established longitudinal cohort known as the Fernald Community Cohort.

This cohort consists of former participants of the Fernald Medical Monitoring Program (FMMP). The FMMP was a voluntary community-based medical surveillance program for residents living within five miles of the perimeter of the former uranium processing plant (Feed Materials Production Center) in Fernald, Ohio for at least a 2-year interval between 1952 and 1984 (Pinney *et al*, 2003; Wones *et al*, 2009). Women who had uranium/radiation exposure, as defined by applying an exposure dosimetry algorithm developed by CDC, were not eligible for this study. Extensive uranium dose reconstruction demonstrated that over 60% of the cohort had such minimal exposure to uranium and radon that their cumulative ionising radiation exposure was less than 3.2% over lifetime background levels (Meyer *et al*, 1996; Killough *et al*, 1998a, 1998b; Pinney *et al*, 2003).

Detailed description of this cohort has been published elsewhere (Wones *et al*, 2009). In summary, upon enrollment in FMMP (starting on 01 December 1990), all participants received a thorough medical examination and diagnostic tests. Periodic medical examinations were offered every 3 years until 1999 and for every 2 years thereafter until 30 November 2008 when the program ended. Female participants age 40 and older were offered mammograms each year. The medical history of the FMMP participants is well characterised and verified; all cancer diagnoses have been validated with medical records.

Extensive disease risk factor information was collected by the FMMP using the initial and the yearly questionnaires. The data on the reproductive history were collected using the baseline FMMP reproductive history questionnaire, the annual reproductive history updates, and the menstrual history questionnaire administered in 2007–2008. History of HRT was established from codes used by the FMMP to characterise the medication taken by the participants throughout the follow-up years that were reported at each exam and on the questionnaire. Family history of breast cancer was available from the questionnaire and physician's history forms. From those data, a total number of relatives with breast cancer, number of first-degree relatives with breast cancer, and the maternal history of breast cancer were extracted for each participant. Each questionnaire asked about the number of drinks per week of different alcoholic beverages. Smoking data were collected at the time of the first exam and annually thereafter using a set of standardised questions from the American Thoracic Society. BMI (kg m^−2^) was calculated from participants' height and weight measured by FMMP staff at the time of each medical exam.

### Assessment of mammographic breast density

Most of the annual FMMP screening mammograms were performed at one of two sites near the Fernald Community, and diagnostic mammograms were conducted primarily at the University of Cincinnati (UC) Medical Center, although some were done at the satellite offices. All the films (both screening and diagnostic) were sent to UC and were read by the same group of Board-certified radiologists (University Radiology Associates) throughout the entire program. Six of those expert radiologists read 70% of all FMMP mammograms over the years. If the woman had a mammogram at another location, she was asked to sign a release for obtaining that mammogram report; these mammograms represented only 2.2% of the mammograms in the database and were not used in this study.

Many FMMP mammograms were performed before 1995 and thus the BI-RADS classification was not included in those mammography reports. However, the radiologist provided a text description of the degree and localisation of breast density on each film. Mammographic reports kept in the FMMP medical charts/database were assigned special FMMP codes from a set of over 230 standardised codes representing specific descriptors in the written mammogram interpretation by the physician, including qualitative breast density assessment. For example, the code 704 (breasts high in density/markedly dense) was assigned to the following description ‘The breast tissue is markedly dense…’. All narrative reports and the corresponding codes were entered into a comprehensive SAS database.

In designing the current study, there were two potential approaches for characterisation of breast density. The level of breast density for each mammogram could be assigned using the radiologist's report (qualitative breast density estimation), resulting in a categorical assessment of density. Alternately, breast density could be evaluated quantitatively using the computer algorithms applied to the digitised images of the mammograms. In order to increase the degree of certainty about the breast density phenotype, a preliminary study of breast density assessment was conducted in the summer of 2007. The details of this study have been described elsewhere (Yaghjyan *et al*, 2011). Briefly, 50 mammograms from three density categories (low (representing BI-RADS I density category), intermediate (representing BI-RADS density categories II and III), high (representing BI-RADS IV density category)) were randomly selected from women eligible for the main study, were re-read by a second Board-certified radiologist (MCM) from the Department of Radiology at UC University Hospital and were scanned with standard equipment (DiagnosticPRO Advantage, VIDAR Systems Corporation, Herndon, VA, USA) to generate digital images. The density group assignment from the second radiologist's reading and the density group assignment from the computer-assisted analysis of digital images (OSIRIS 4 Software, Geneva, Switzerland) were compared against the original breast density assignment (from FMMP mammography codes). In this preliminary study, substantial to perfect agreement was observed between density categories as determined from two radiologists' readings (kappa statistic (*κ*)=0.73), but agreement between radiologist's reading and density assignment from digitised images was fair (*κ*=0.27). When analysis was restricted to extreme density categories (low and high), the agreement between radiologists was perfect (*κ*=0.81), whereas agreement between radiologist's reading and density assignment from digitised images did not improve (*κ*=0.24) (Yaghjyan *et al*, 2011). Based on these findings, we chose to use the radiologist's reading for characterisation of breast density rather than using information from digitised radiography films (*κ* statistic 0.81 *vs* 0.24).

### Study design

To be included in this nested case–control study of high and low breast density phenotypes, women were required to have been enrolled in FMMP as adults, and have had at least one mammogram in the FMMP mammography database. Out of 3832 women age-eligible for screening mammograms and having at least one study, 84% had more than one mammogram and more that 50% of women had seven mammograms or more. The median time between the first and last mammogram, for the 3217 women with more than one, was 12.26 years (range 0.05–18.03 years). As the cohort is >99% Caucasian, participants of other races were not included. A woman was not eligible for the study if she had a history of breast cancer, genetic syndromes/disorders with underdevelopment of breast/small stature (ICD-9 codes 259.4, 253.3, 758.6, 752.7), a history of mammoplasty or was transsexual.

We used a case–control sampling frame to identify women with dense breasts (high-density phenotype) and women who never had high or moderate breast density on any of their mammograms in FMMP database, that is, have sustained low density on all mammograms throughout the cohort follow-up (low-density phenotype). High and low breast densities were defined using the FMMP mammogram coding system and these code criteria were applied to all of the mammograms of the eligible women in the database in order to establish their life-long density history rather than using a single mammogram. Women with high breast density had high density on *any* of their mammograms in the FMMP database (FMMP code 704) and also had at least one of the 14 additional codes. These additional codes represented a set of codes from the mammogram coding system that were assigned to high-density mammograms by the two radiologists (original FMMP reading and the second radiologist's reading from the preliminary study) and therefore indicated a more precise assignment of high density. High breast density was equivalent to BI-RADS category IV (extremely dense). Women who had no codes that would indicate high or intermediate density on *any* of their mammograms in the FMMP database were included in the low breast density group. In addition, the low-density mammogram report had to contain one of the descriptors indicative of a ‘normal’ mammogram (normal breast, negative; no signs of breast carcinoma/no suspicious abnormality; routine screenings; follow-up mammogram in 2 years; follow-up in 5 years). Low density was equivalent to BI-RADS category I (fatty breasts).

An index (reference) mammogram for a woman was selected from all of her mammograms if she met high- or low-density definition. The index mammogram was required to have FMMP codes used to define the density phenotype, be a screening (93% of the mammograms) or diagnostic (7% of the mammograms) study, and be a part of FMMP medical examinations. The woman was required to be 40–80 years old at the time of the index mammogram and to have a BMI<35 at the time of the mammogram, to exclude a few morbidly obese women, as BMI⩾35 has been consistently reported to be associated with lower density (Sala *et al*, 1999; Gapstur *et al*, 2003; Titus-Ernstoff *et al*, 2006; Irwin *et al*, 2007). As the result, we excluded 24 women with high density and 79 women with low density. Morbid obesity is often associated with other pathological conditions and underlying biological processes may have potential to influence breast density and/or interact with other breast cancer risk factors in unknown ways. Exclusion of morbidly obese women provides a more representative sample of women and removes potential for those effects.

The earliest mammogram that met the above criteria was chosen as the index mammogram. Of the 3594 mammograms for 356 women with high density, 265 mammograms met the index mammogram criteria. Of the 6873 mammograms for 1148 women with low density, 860 mammograms met the index mammogram criteria.

The study was conducted under the FMMP IRB protocol (04-02-07-05-EE, last approved by the University of Cincinnati Institutional Review Board on 1 December 2011).

### Statistical analysis

Statistical analyses were done using SAS software (version 9.2, SAS Institute, Cary, NC, USA). The relationship between the potential predictors and the breast density phenotype was examined using logistic regression. Variables in the analysis included age and BMI at index mammogram date, reproductive history, smoking, alcohol, HRT, and a family history of breast cancer. For variables cumulative in nature (smoking, alcohol use, HRT), the information was extracted from all the questionnaires completed before the index mammogram (reference) date. Information on reproductive history and family history of breast cancer was obtained from the last completed questionnaire available from before/on the reference mammogram date. Menopausal status at the time of the index mammogram was determined from the annual reproductive history updates and the menstrual history questionnaire completed before the date of the index mammogram. Age was modeled as a categorical rather than continuous variable, as the relationship between age and breast density is not linear and changes with menopausal transition.

Parity was defined as the number of pregnancies that resulted in single, multiple birth, or stillbirth with ⩾34 weeks of gestation. In an adjusted logistic regression analysis, parity and age at first child's birth were modeled as categorical with three levels (parity 0, 1–2, ⩾3; age at first child's birth <20, 20–29, ⩾30). Nulliparous women had no value for the age at first child's birth (73 low density, 33 high density) and were included in the category ⩾30. Menopausal status at mammogram and HRT were represented by one variable with three levels (premenopausal women, postmenopausal women who never used HRT, postmenopausal women with a history of HRT). We did not separate current and past HRT users as the small numbers of women in some subsets did not provide sufficient power to draw meaningful conclusions. Alcohol use, smoking, and HRT were modeled as dichotomous variables (ever used *vs* never). BMI at mammogram was categorised as <25, 25–29, ⩾30. Age was modeled as categorical in all the analysis (40–49, 50–59, 60–69, ⩾70), as this approach provided a better model fit compared to continuous age.

Missing values for the age at menarche (7 low density, 4 high density), cumulative pack years of smoking (3 low density, 3 high density), current number of drinks per week (20 low density, 5 high density), and cumulative number of drinks per week (14 low density, 2 high density) were substituted with the median value for women with low density.

A multi-step approach was used to impute values for the missing menopausal status and the age at menopause (Kroke *et al*, 2001). Information on the menopausal status and age at menopause was first obtained using direct questions from the FMMP questionnaires and the Menstrual History Questionnaire. For all women with missing information (67 low density, 5 high density), the median age at menopause in women with low density who had reported natural menopause (50 years) was used as a cut-off to determine the woman's menopausal status.

After the logistic regression with all women in the study, the analysis was stratified by menopausal status at mammogram, HRT history, and a family history of breast cancer. The risk estimates are presented as ORs and their corresponding 95% confidence intervals (CI). Statistical difference in subset-specific risk estimates were determined using test of heterogeneity (statistical interaction). Significance in all the analyses was assessed at 0.05 level. For all of the presented models, Hosmer–Lemeshow test of goodness of fit indicated reasonable model fit (*P*>0.05).

## Results

### Distribution of breast density risk factors in the study population

This nested case–control study included 265 women with high and 860 women with low breast density. The distribution of breast density risk factors in the study population is presented in Table 1. Two density groups were similar with respect to the age at mammogram, age at menarche, menopausal status, and a family history of breast cancer. Compared with women with low density, women with high density had a lower BMI at mammogram, were younger at menopause, were older at the first child's birth, and had a larger proportion of women with fewer children and women with a history of HRT. Women with high density were also more likely to be alcohol users and less likely to be smokers compared with women with low density. For the women in this nested case–control study, the median length of time between mammograms, for the 980 women who had more than one, was 12.03 years, range 0.06–18.09 years.

### Associations of breast health risk factors with breast density

The results of the adjusted logistic regression are presented in Tables 2 and 3. In the overall analysis, breast density was inversely associated with BMI (*P* for trend=0.02) and parity (*P* for trend <0.001). Postmenopausal women with a history of HRT had increased breast density compared with premenopausal women, but this association was only marginally significant (OR 1.4, 95% CI 1.0–2.1). Women with a history of alcohol use were at a greater risk of increased breast density (OR 2.0, 95% CI 1.4–2.8).

In premenopausal women, breast density was inversely associated with BMI (*P* for trend<0.01) and was positively associated with alcohol consumption (OR 2.2, 95% CI 1.4–3.5) (Table 2). In postmenopausal women, breast density was inversely associated with parity (*P* for trend<0.001) and positively associated with HRT history (OR=2.1, 95% CI 1.4–3.3) and alcohol consumption (OR=1.9, 95% CI 1.2–3.0). We observed no significant association of BMI with breast density in postmenopausal women (*P* for interaction between BMI and menopausal status=0.08).

In women with a history of HRT, breast density significantly increased with age (*P* for trend <0.001) and alcohol use (OR=3.6, 95% CI 1.7–7.7) and decreased with larger parity (*P* for trend <0.001) (Table 3). In the subset of women who never used HRT, breast density was inversely associated with age (*P* for trend<0.001), BMI (*P* for trend <0.01), and parity (*P* for trend=0.02), and was positively associated with alcohol consumption (OR 1.6, 95% CI 1.1–2.4). The associations of parity and alcohol consumption with density in this subset were less strong than those associations in women with HRT history (*P* for interaction <0.001 for both variables).

In women with a family history of breast cancer, breast density was positively associated with alcohol use (OR=3.7, 95% CI 1.7–7.8) (Table 3). Similar, but weaker association of alcohol consumption with density was seen in women without a family history of breast cancer (OR=1.7, 95% CI 1.2–2.5, *P* for interaction<0.001). Among women with no family history of breast cancer, breast density was inversely associated with parity (*P* for trend <0.001). In this subset, the risk of high breast density was marginally increased in postmenopausal women with a history of HRT as compared with premenopausal women (OR 1.5; 95% CI 1.2–2.6).

## Discussion

This retrospective study of 265 women with high breast density and 860 women with low breast density was designed to explore differences in the direction and strength of associations between the potential predictors of breast density and the breast density phenotype. Analyses were conducted in subsets of women based upon their menopausal status, history of postmenopausal hormone use, and a family history of breast cancer.

Using the FMMP population had several advantages. The extensive and detailed exposure estimation done by CDC allowed the selection of women who were unexposed to uranium and radiation for this study. The FMMP database contains comprehensive information on breast cancer risk factors collected prospectively over an 18-year period using standardised questionnaires and medical examinations. The extensive FMMP mammography database (31 043 mammograms) allowed defining the breast density phenotype based on the life-long density history for each woman rather than by a single mammogram. Mammograms were carefully coded using the FMMP coding system; however, there is a possibility of inter-observer variation in assessment of the density, as the films were evaluated by different radiologists. To overcome this limitation, the results of the preliminary study were used to improve definition of the breast density phenotype. Prospective data collection and sampling of the extreme breast density phenotypes allowed assessment of breast density predictors in an efficient design.

Of the 2168 women eligible for the study, only 1504 (69%) met the definition for either high or low-density study group. Selection of women with extreme density categories increases certainty about the outcome and removes chances for density misclassification. Inter-rater agreement in density assessment has been reported to be substantial to perfect within a given density assessment method regardless of the assessment approach, and was the best for the extreme density patterns (Ciatto *et al*, 2005; Martin *et al*, 2006; Nicholson *et al*, 2006; Ooms *et al*, 2007; van de Ven *et al*, 2008; Tagliafico *et al*, 2009; Garrido-Estepa *et al*, 2010). Such a selection also increases statistical power (Guey *et al*, 2011). However, the findings might not apply to women with intermediate density patterns. Finally, due to the lack of racial heterogeneity of the FMMP cohort (99% White-non-Hispanic), the findings are limited to one racial group.

Consistent with previous reports (Gram *et al*, 1995; Sala *et al*, 1999; El-Bastawissi *et al*, 2000; Lam *et al*, 2000; Vachon *et al*, 2000, 2002, 2005; Colacurci *et al*, 2001; Erel *et al*, 2001; Gapstur *et al*, 2003; Warwick *et al*, 2003; Conner *et al*, 2004; Maskarinec *et al*, 2006; Modugno *et al*, 2006; Noh *et al*, 2006; Titus-Ernstoff *et al*, 2006; Johansson *et al*, 2008; Kelemen *et al*, 2008), we found an inverse relationship between BMI and breast density, but these findings were significant only in premenopausal women of our study. BMI affects breast density by different mechanisms. In premenopausal women, increased BMI has been linked to decreased levels of estradiol (Onland-Moret *et al*, 2005), a potent stimulator of epithelial and stromal proliferation in the breast tissue (Pike *et al*, 1993; Foidart *et al*, 1998; Johansson *et al*, 1998; Sutherland *et al*, 1998; Henderson and Feigelson, 2000; Seeger *et al*, 2004; Russo *et al*, 2006; LaMarca and Rosen, 2007; Pattarozzi *et al*, 2008). Adipose tissue looks radiolucent on the mammogram and creates an image of less dense breast tissue. The net effect of BMI on breast density depends on the relative contribution of these mechanisms.

The inverse association of parity with breast density observed in this analysis was consistent with the previous reports (Gram *et al*, 1995; Noh *et al*, 2006). We found for the first time statistically significant interaction between parity and postmenopausal hormone use. The inverse association of parity with breast density could be explained by biological changes that take place in the breast tissue during a full-term pregnancy that lead to permanent gene expression changes in type 3 lobules making them less susceptible to hormonal influences and carcinogenesis (Russo and Russo, 1995; Russo *et al*, 2005). As a result, the cell-proliferative effect of estrogen on the breast tissue in parous women could be less prominent than in nulliparous women leading to a lesser degree of density. It is possible that additional hormonal stimulation of type 1 and type 2 lobules by exogenous hormones (HRT) in nulliparous women could have a stronger effect on breast epithelium proliferation compared with parous women with a larger proportion of hormone-resistant type 3 lobules, and could explain a stronger association of parity with breast density in women with a history of HRT.

Our findings indicated that breast density increased with age in older women with HRT history but decreased in those who never used HRT. These findings are consistent with the previously reported lower density in older (postmenopausal) women and an increased density in HRT users (Gapstur *et al*, 2003; Titus-Ernstoff *et al*, 2006; Kelemen *et al*, 2008). The positive association of age with breast density in HRT users could reflect the longer cumulative exposure to exogenous hormones, whereas the inverse association of age to breast density in women who never used HRT could reflect the decline in estradiol levels with age. In this analysis, postmenopausal women with a history of HRT were more likely to have denser breasts compared with premenopausal women; these findings were consistent with other reports (Rutter *et al*, 2001; Vachon *et al*, 2002; Gapstur *et al*, 2003; Titus-Ernstoff *et al*, 2006; Kelemen *et al*, 2008). The hormonal environment appears to be one of the important regulators of breast density. Postmenopausal hormones, especially combined estrogen-progesterone preparations, cause breast epithelium to proliferate at a higher rate, and thus, increase breast density.

The association between alcohol use and breast density has been reported (Vachon *et al*, 2000; Maskarinec *et al*, 2006). We reported for the first time a stronger effect of alcohol in women with a family history of breast cancer, which could suggest a gene-environment interaction. Continuous stimulation of aromatase activity by alcohol could cause an increase in active estrogen levels in the peripheral tissues, including the breast, which could result in an increased breast epithelium proliferation (Zhu and Conney, 1998; Onland-Moret *et al*, 2005).

Our findings suggested that BMI, parity, HRT, and alcohol consumption appear to be important predictors of breast density in different subsets of women. Some associations differ by family history of breast cancer and HRT history. When possible, reduction in breast density by limiting alcohol and HRT not only reduces the risk of developing breast cancer but also facilitates an early detection of breast cancer in the less dense breast tissue.

## Figures and Tables

**Table 1:** Characteristics of the study population by low or high breast density

**Characteristic**	**Women with low density (*N*=860)**	**Women with high density (*N*=265)**
Age at mammogram (years), mean (s.d.)	51.2 (10.1)	51.5 (9.3)
		
*Age group, N* (%)		
40–49	466 (54.2)	133 (50.2)
50–59	213 (24.8)	82 (30.9)
60–69	126 (14.7)	40 (15.1)
⩾70	55 (6.4)	10 (3.8)
		
BMI at mammogram, mean (s.d.)^a^	26.9 (4.1)	26.1 (4.2)
		
*BMI category, N* (%)^a^		
<25	304 (35.4)	120 (45.3)
25–29	348 (40.5)	92 (34.7)
30–34	208 (24.2)	53 (20.0)
		
Age at menarche (years), mean (s.d.)	12. 8 (1.5)	12.8 (1.4)
Age at first child's birth (years), mean (s.d.)^a^	22.6 (4.5)	23.8 (4.7)
		
*Age at first child's birth* a		
<20	237 (27.6)	48 (18.1)
20–29	490 (57.0)	161 (60.8)
⩾30	131 (15.2)	54 (20.4)
		
*Parity, N* (%)^a^		
No children	73 (8.5)	33 (12.5)
Parity 1–2	360 (41.9)	145 (54.2)
Parity ⩾3	425 (49.4)	85 (32.1)
		
*Menopausal status, N* (%)		
Premenopausal	405 (47.1)	120 (45.3)
Postmenopausal	455 (52.9)	145 (54.7)
		
Age at menopause (years), mean (s.d.)^a^	48.1 (7.9)	44.6 (7.3)
Ever consumed alcohol (yes), *N* (%)^a^	158 (18.4)	82 (30.9)
Alcohol consumption at the time of the mammogram (yes), *N* (%)^a^	149 (17.3)	63 (23.8)
Ever smoked, (yes), *N* (%)	378 (44.0)	108 (40.8)
Smoking at the time of the mammogram (current pack years), mean (s.d.)^a^	5.7 (12.8)	0.2 (0.7)
		
*Family history of breast cancer*		
First degree relatives with		
BC, *N* (%)		
0	777 (90.4)	248 (93.6)
⩾1	83 (9.7)	17 (6.4)
Any degree relatives with BC, *N* (%)		
0	674 (78.4)	207 (78.1)
1	148 (17.2)	44 (16.6)
⩾2	38 (4.4)	14 (5.3)
		
Hormone replacement therapy (Ever), *N* (%)^a^	193 (22.4)	88 (33.2)
		
*Menopausal status and HRT history combined* a		
Premenopausal, no HRT	405 (47.1)	120 (45.3)
Postmenopausal, no HRT	262 (30.5)	57 (21.5)
Postmenopausal, used HRT	193 (22.4)	88 (33.2)

a Significant difference between women with high and low density at 0.05 level.

**Table 2:** Results of adjusted logistic regression for the association between breast cancer risk factors and high breast density, stratified by menopausal status

**Covariate**	**All women in the study 263 high/858 low density**	**Pre-menopausal women 118 high/404 low density**	**Post-menopausal women 145 high/454 low density**
*Age at mammogram*			
40–49	1.0	1.0	1.0
50–59	1.7 (1.2–2.5)	1.3 (0.7–2.4)	2.1 (1.2–3.7)
60–69	1.6 (0.9–2.6)	NA^a^	1.8 (1.0–3.2)
⩾70	1.0 (0.4–2.2)	NA^a^	1.1 (0.5–2.7)
			
*BMI at mammogram*			
<25	1.0	1.0	1.0
25–29	0.7 (0.5–1.0)	0.6 (0.4–1.0)	0.8 (0.5–1.3)
30–34	0.7 (0.5–1.0)	0.4 (0.2–0.8)	1.0 (0.6–1.6)
			
Age at menarche	1.0 (0.9–1.1)	1.0 (0.84–1.1)	1.1 (0.9–1.2)
			
*Age at first child's birth*			
<20	0.9 (0.5–1.7)	0.9 (0.4–1.9)	1.0 (0.4–2.4)
20–29	1.0	1.0	1.0
⩾30	0.7 (0.5–1.0)	0.9 (0.5–1.6)	0.5 (0.3–0.9)
			
*Parity*			
0	2.0 (0.9–4.0)	2.0 (0.7–5.5)	1.9 (0.6–5.9)
1–2	2.1 (1.5–2.9)	1.5 (0.9–2.4)	2.7 (1.7–4.1)
⩾3	1.0	1.0	1.0
			
*Menopausal status/HRT use*			
Pre	1.0	NA^b^	NA^b^
Post, used HRT	1.4 (1.0–2.1)	NA^b^	2.1 (1.4–3.3)
Post, no HRT use	0.7 (0.4–1.1)	NA^b^	1.0
			
Ever smoked (yes vs no)	0.9 (0.6–1.2)	0.7 (0.5–1.2)	1.0 (0.6–1.4)
Ever consumed alcohol (yes vs no)	2.0 (1.4–2.8)	2.2 (1.4–3.5)	1.9 (1.2–3.0)
Maternal history of breast cancer (yes vs no)	0.5 (0.2–1.1)	0.3 (0.1–1.0)	0.8 (0.3–2.2)
Total number of relatives with breast cancer	1.1 (0.9–1.5)	1.4 (0.9–2.1)	1.0 (0.6–1.5)

a Not available; age groups are not represented in the subset of premenopausal women.

b Not applicable; the variable is used to create the subsets by menopausal status and by HRT use history.

**Table 3:** Results of adjusted logistic regression for the association between breast cancer risk factors and the risk of high breast density phenotype, stratified by hormone replacement therapy use and a family history of breast cancer

**Covariate**	**Women with history of HRT 88 high/193 low density**	**Women without history of HRT 174 high/658 low density**	**Women with FamHx^a^ of breast cancer 57 high/185 low density**	**Women without FamHx^a^ of breast cancer 206 high/673 low density**
*Age at mammogram*				
40–49	1.0	1.0	1.0	1.0
50–59	5.2 (2.3–11.6)	1.0 (0.6–1.6)	2.9 (1.2–7.5)	1.6 (1.0–2.4)
60–69	11.4 (4.6–28.2)	0.3 (0.1–0.6)	1.9 (0.6–6.4)	1.5 (0.8–2.6)
⩾70	16.2 (3.1–85.0)	0.2 (0.1–0.7)	1.0 (0.1–10.3)	1.0 (0.4–2.3)
				
*BMI at mammogram*				
<25	1.0	1.0	1.0	1.0
25–29	0.8 (0.4–1.6)	0.6 (0.4–0.9)	0.5 (0.3–1.2)	0.7 (0.5–1.0)
30–34	1.4 (0.6–3.2)	0.5 (0.3–0.8)	0.7 (0.3–1.7)	0.7 (0.4–1.1)
				
Age at menarche	1.1 (0.9–1.4)	1.0 (0.9–1.1)	0.9 (0.7–1.2)	1.0 (0.9–1.2)
				
*Age at first child's birth*				
<20	2.2 (0.5–9.7)	0.9 (0.5–1.8)	1.1 (0.3–3.7)	0.8 (0.4–1.6)
20–29	1.0	1.0	1.0	1.0
⩾30	0.5 (0.2–1.0)	0.7 (0.5–1.1)	0.5 (0.2–1.2)	0.7 (0.5–1.0)
				
*Parity*				
0	1.8 (0.3–0.4)	1.5 (0.6–3.7)	2.1 (0.4–10.5)	1.9 (0.8–4.5)
1–2	4.2 (2.1–8.4)	1.7 (1.2–2.5)	1.9 (0.9–4.0)	2.1 (1.5–3.1)
⩾3	1.0	1.0	1.0	1.0
				
*Menopausal status*				
Pre	NA^b^	1.0	1.0	1.0
Post, used HRT	NA^b^	NA^b^	1.1 (0.4–2.9)	1.5 (1.0–2.4)
Post, no HRT use	NA^b^	1.5 (0.9–2.5)	0.3 (0.1–1.1)	0.8 (0.5–1.4)
				
Ever smoked (yes vs no)	0.8 (0.4–1.5)	0.8 (0.6–1.2)	1.3 (0.6–2.5)	0.8 (0.6–1.1)
Ever consumed alcohol (yes vs no)	3.6 (1.7–7.7)	1.6 (1.1–2.4)	3.7 (1.7–7.8)	1.7 (1.2–2.5)
Maternal history of breast cancer (yes vs no)	0.8 (0.2–3.9)	0.5 (0.2–1.2)	0.5 (0.2–1.1)	NA^c^
Total number of relatives with breast cancer	0.9 (0.5–1.7)	1.1 (0.8–1.6)	NA^d^	NA^d^

a Family history.

b Not applicable; the variable is used to create the subsets by menopausal status and by HRT use history

c Not applicable; women without family history of breast cancer by definition had no maternal history of breast cancer.

d Not applicable; the variable is used to create the subsets by family history of breast cancer.
